# Mesoporous Confinement of Fluorescent Dyes in Ultra-Transparent Silica Aerogel Films via Tailored Sol–Gel Kinetics

**DOI:** 10.3390/gels12080676

**Published:** 2026-07-30

**Authors:** Zhizhong Qin, Yuntao Li, Guifeng Wang, Fengyu Li, Pengchao Song, Xihao Sun, Yong Jiang, Jialu Lu, Wei Wei

**Affiliations:** 1 Lily Group Co., Ltd., No. 1768, Jingwu Road, Qiantang District, Hangzhou 311200, China; 2 School of Chemistry and Chemical Engineering, School of Automotive and Traffic Engineering, Public Experiment and Service Center, Jiangsu University, Xuefu Road 301, Zhenjiang 212013, China; 3 Shanghai Key Laboratory of Special Artificial Microstructure and Technology, School of Physics Science and Engineering, Tongji University, Siping Road 1239, Shanghai 200092, China

**Keywords:** sol–gel kinetics, mesoporous confinement, host–guest interaction, fluorescence blue shift, silica aerogel films

## Abstract

Silica aerogel films are highly promising matrices for advanced optical applications, yet balancing ultra-high transmittance with structural stability during functionalization remains a critical challenge. Directly incorporating organic dyes often leads to aggregation and severe photodegradation, necessitating a robust host–guest encapsulation strategy. Herein, we report the fabrication of ultra-transparent, fluorescent silica aerogel films via precisely tailored acid/base two-step sol–gel kinetics and dip-coating. The optimized pure silica matrix achieves a peak visible transmittance of 97.4% and sub-nanometer surface smoothness (RMS = 276.7 pm). By utilizing this pristine network, Rhodamine 6G (Rh6G) and Rhodamine B (RhB) dyes were effectively confined within the amorphous mesoporous pores. Notably, RhB exhibited superior matrix integration, indicated by an H4 hysteresis loop transition and a significantly reduced pore volume (0.019 cm^3^/g). This mesoporous confinement successfully suppressed dye quenching, prolonging the fluorescence lifetimes to 5.22 ns and 5.36 ns for Rh6G and RhB, respectively. Crucially, we elucidate that the electrostatic and hydrogen-bonding interactions between the silica pore walls and the dye’s xanthene rings elevate the excited-state energy, inducing a distinct matrix-driven emission blue shift. This work provides a scalable pathway for high-performance optical coatings and offers deep insights into host–guest interfacial coupling in gel networks.

## 1. Introduction

Silica aerogel films, characterized by their high porosity, tunable refractive index, and robust three-dimensional network, have emerged as highly promising platforms for advanced optical applications, ranging from broadband antireflection coatings to photonic sensors [1,2,3]. The traditional sol–gel fabrication of these films typically relies on either acid- or base-catalyzed routes [4,5,6]. However, an inherent trade-off exists: acid-catalyzed gels often suffer from poor optical transmittance due to low cross-linking degrees, whereas base-catalyzed particulate networks exhibit compromised mechanical integrity [7,8]. Consequently, precisely tuning the sol–gel gelation kinetics via an acid/base two-step strategy is imperative to construct an ultra-smooth, highly transparent matrix without sacrificing its robust mesoporous skeleton.

Beyond passive optical transmission, integrating highly emissive organic molecules, such as xanthene dyes (e.g., Rhodamine 6G and Rhodamine B), into the silica matrix presents a viable pathway toward active luminescent materials. Nevertheless, direct incorporation of organic dyes frequently encounters severe challenges, including dye aggregation, phase separation, and rapid photodegradation under continuous excitation, which drastically deteriorate both the optical transparency and the fluorescence stability of the films [9,10,11]. To overcome this limitation, exploiting the rigid, mesoporous silica network for the effective host–guest confinement and encapsulation of dye molecules is highly desirable to shield them from environmental quenching.

While mesoporous confinement effectively stabilizes the encapsulated fluorophores [12], the intrinsic physical-chemical interactions at the organic-inorganic interface remain largely enigmatic [13]. Most current studies phenomenologically report the preservation of luminescence, yet there is a critical lack of mechanistic insight into how the surface functionalities of the silica pore walls (e.g., silanol groups and electronegative oxygen ions) fundamentally interact with the conjugated aromatic rings of the dyes [14,15]. Consequently, understanding how these interfacial interactions alter the photophysical properties and excited-state energy of the confined molecules is still a significant challenge [16]. Elucidating this matrix-induced emission mechanism is crucial for the rational design of multifunctional aerogel films.

In this work, we achieve the precise construction of ultra-transparent and highly fluorescent silica aerogel films via a tailored acid/base two-step sol–gel strategy. The primary scientific achievement of this study is the successful decoupling of the hydrolysis and condensation kinetics, which intrinsically prevents macroscopic phase separation and yields a robust mesoporous network with sub-nanometer smoothness and an exceptional peak visible transmittance. Leveraging this rigid ‘optical container’, we further achieved the deep mesoporous confinement of xanthene dyes (Rh6G and RhB). Crucially, rather than merely reporting the optical phenomena, we systematically elucidate the host–guest photophysical dynamics: we demonstrate that the robust electrostatic and hydrogen-bonding interactions—particularly enhanced by the free carboxyl group of RhB—prolong fluorescence lifetimes, and induce a matrix-driven emission blue shift. Ultimately, these achievements not only provide a scalable methodology for fabricating high-performance optical coatings but also establish a fundamental structural paradigm for manipulating host–guest interactions within confined gel networks.

## 2. Results and Discussion

### 2.1. Sol–Gel Transition Kinetics and Silica Network Evolution Under Acid/Base Dual Catalysis

The construction of a mesoporous silica matrix with both ultra-high optical transmittance and robust mechanical stability relies fundamentally on precisely tuning the hydrolysis and condensation kinetics of the tetraethyl orthosilicate (TEOS) precursor. This kinetic control dictates the cross-linking degree of the underlying siloxane skeleton, profoundly impacting the optimized gelation time properties of the sol and the subsequent film quality [17].

**Figure 1:**
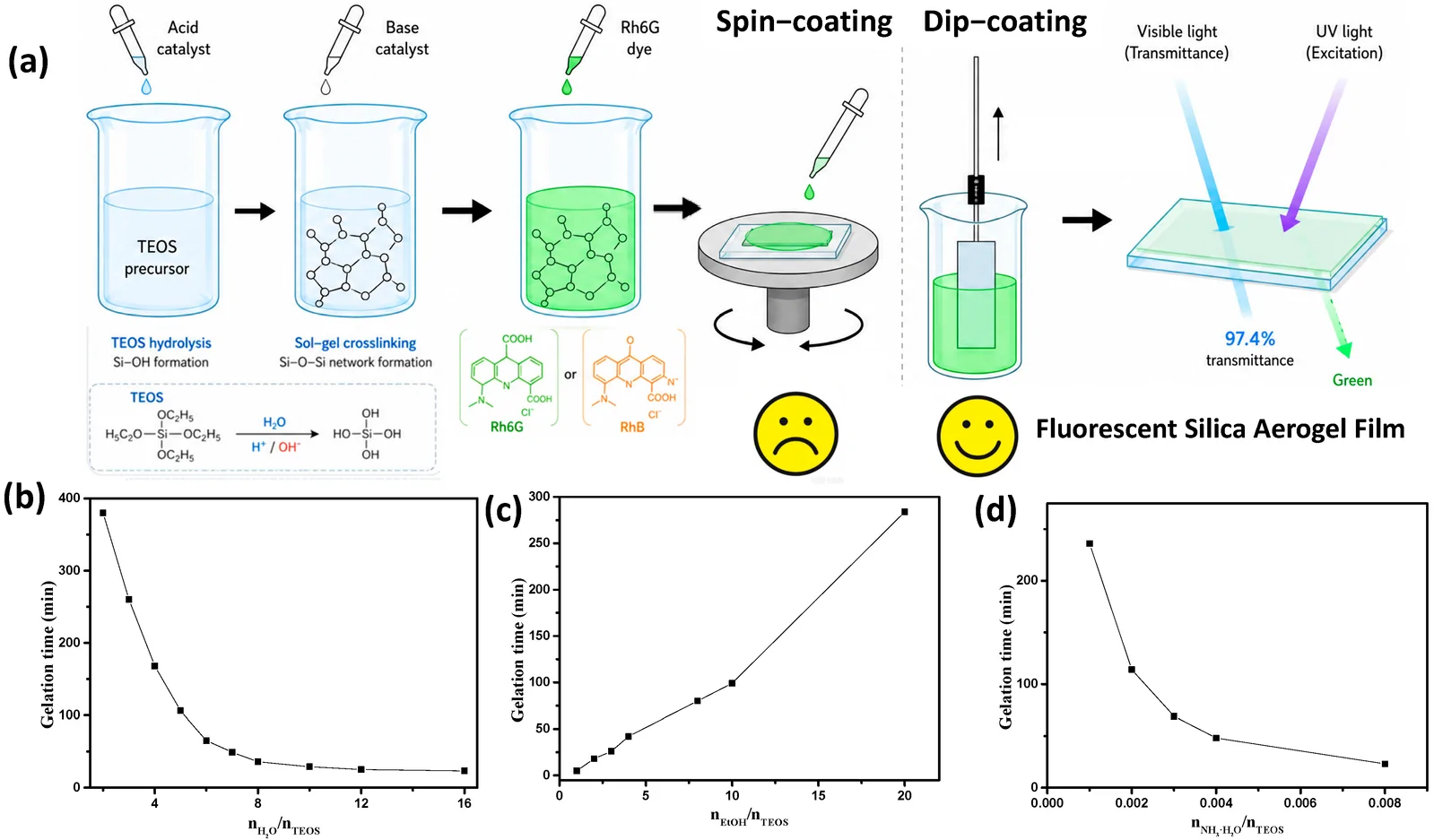
Schematic illustration of the acid/base two-step sol–gel process and the gelation kinetics tuning for optimal film fabrication. (**a**) Flow chart detailing the preparation of the highly transparent and fluorescent silica aerogel films via spin- or dip-coating. Dependence of gelation time on the molar ratios of (**b**) H_2_O/TEOS, (**c**) EtOH/TEOS, and (**d**) NH_3_·H_2_O/TEOS.

As illustrated in Figure 1a, an acid/base two-step sol–gel process was designed to fabricate the aerogel films. Within this dual-catalytic system, the sol-to-gel transition (gelation time) exhibits high sensitivity to the molar ratios of the reactive components. Figure 1b–d systematically elucidates the effects of the molar ratios of H_2_O/TEOS, EtOH/TEOS, and NH_3_·H_2_O/TEOS on the overall gelation kinetics.

During the initial acid-catalyzed stage, oxalic acid effectively promotes the hydrolysis of TEOS, generating abundant linear siloxane oligomers [6,18]. In the subsequent stage, the introduction of ammonia (base catalyst) rapidly triggers the condensation reaction, leading to a sharp increase in the network cross-linking degree [19]. As shown in Figure 1d, a slight increase in the NH_3_·H_2_O/TEOS molar ratio from 0.001 to 0.008 significantly accelerates condensation, drastically reducing the gelation time from 236 min to 24 min. Furthermore, water acts as both a key reactant in hydrolysis and a byproduct of condensation [20]; increasing its ratio (nH_2_O/nTEOS from 2 to 16) concurrently accelerates the reaction rate, decreasing the gelation time from 380 min to 23 min (Figure 1b). Conversely, ethanol serves as the solvent for dispersing the silica precursors [21,22]. Increasing the ethanol content dilutes the system, thereby effectively retarding the cross-linking and solidification of the gel network (Figure 1c) [23].

Crucially, an excessively rapid gelation rate induces severe agglomeration of silica particles, resulting in a heterogeneous network that compromises the optical transmittance of the film [24]. Conversely, excessively slow gelation fails to provide a suitable viscosity essential for uniform film deposition [25]. Through systematic optimization of these multidimensional kinetic parameters, the final precursor molar ratio was precisely determined as 1:7:4:0.001:0.003 (TEOS:H_2_O:EtOH:H_2_C_2_O_4_:NH_3_·H_2_O).

This tailored composition successfully restricts the optimized gelation time to approximately 1 h. Such an optimal kinetic timeframe not only ensures the robust development of the amorphous Si-O-Si cross-linked network but also guarantees excellent tailored gelation kinetics for the subsequent dip-coating process. Consequently, it lays a solid structural foundation for achieving ultra-high optical transmittance and sub-nanometer surface smoothness in the resulting films. The relevant physicochemical properties of the silica-based aerogel films are shown in Table 1.

### 2.2. Morphological Superiority and Optimization for Peak Transmittance

**Figure 2:**
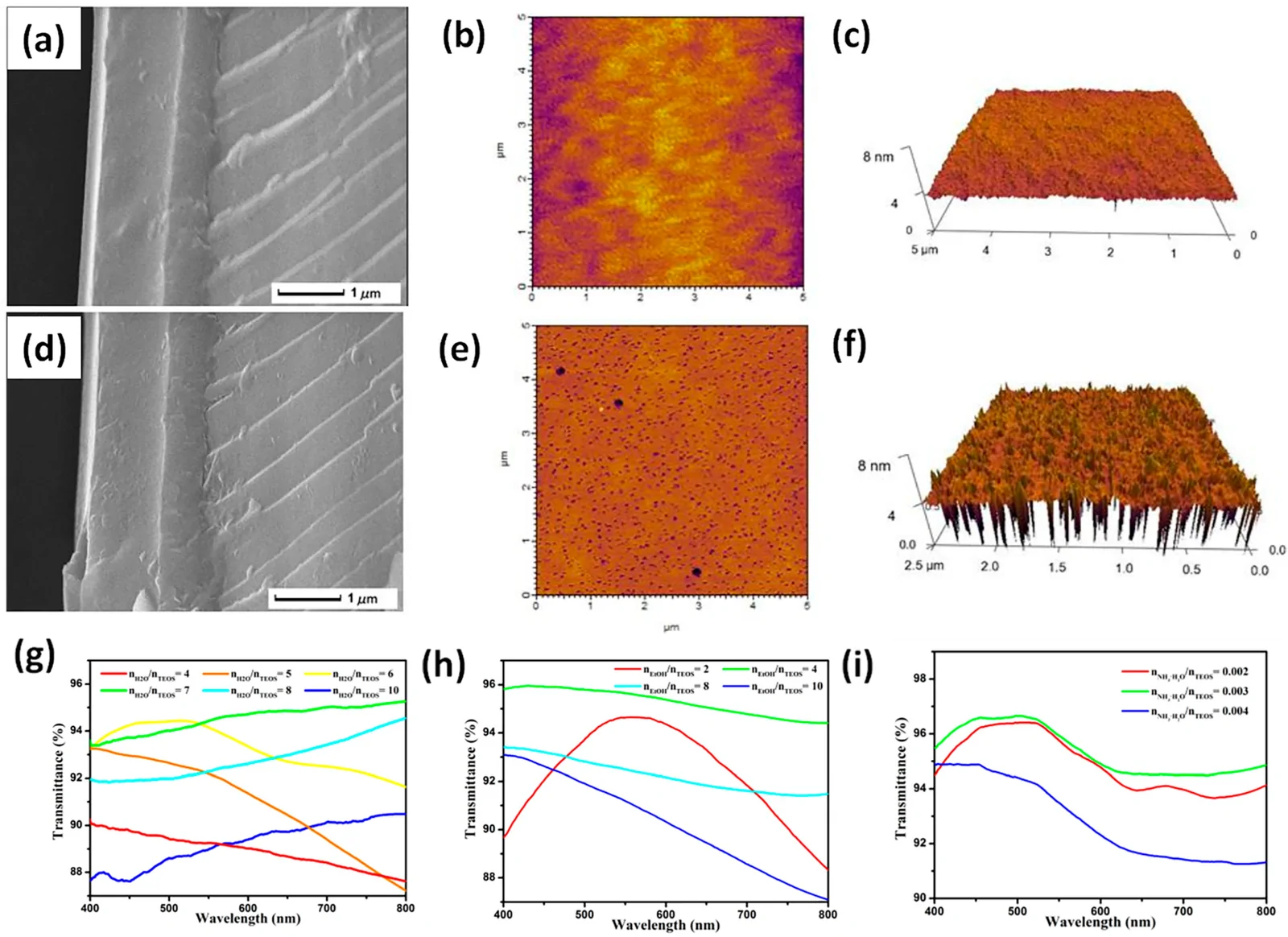
Morphological superiority of the dip-coating method and the optimization of sol–gel parameters for ultra-high transmittance. Cross-sectional SEM images of silica aerogel films prepared by (**a**) dip-coating and (**d**) spin-coating. Corresponding 2D and 3D AFM topographic images of films prepared by (**b**,**c**) dip-coating and (**e**,**f**) spin-coating. UV-Vis transmittance spectra of the dip-coated films as a function of the molar ratios of (**g**) H_2_O/TEOS, (**h**) EtOH/TEOS, and (**i**) NH_3_·H_2_O/TEOS.

To elucidate the deterministic impact of the film-forming process on the micro-morphology of the aerogel films, we systematically compared the pure silica films prepared by dip-coating and spin-coating (Figure 1a). Superior macroscopic optical transmittance is fundamentally rooted in the ultra-low surface roughness and dense internal cross-section of the films. Comparative analysis using scanning electron microscopy (SEM, Figure 2a,d) and atomic force microscopy (AFM, Figure 2b,c,e,f) reveals that, compared to the spin-coating method (root-mean-square, RMS = 999.5 pm), the dip-coating process exhibits pronounced morphological superiority, achieving sub-nanometer surface smoothness (RMS = 276.7 pm, R_a_ = 224.8 pm). 

This is primarily attributed to the intense centrifugal forces during the spin-coating process, which trigger rapid and non-uniform solvent evaporation, consequently inducing stress concentration and micro-fluctuations within the fragile siloxane gel network [26]. In stark contrast, the dip-coating method demonstrated pronounced morphological superiority. During the steady withdrawal process, driven by the synergistic effects of capillary forces and gravity, the gradual solvent evaporation allows the siloxane network to undergo sufficient stress relaxation and homogeneous cross-linking on the substrate [27,28]. Following the selection of the dip-coating technique, Figure 2g–i systematically elucidates the profound impact of compositional fine-tuning on the ultimate UV-Vis transmittance spectra. This ultra-smooth and uniform surface effectively suppresses interfacial light-scattering losses, serving as the crucial physical foundation for the subsequent realization of the peak optical transmittance of 97.4%.

Conversely, this ultra-smooth and dense structural finish effectively minimizes interfacial light-scattering losses [29,30]. Subsequent compositional optimization reveals that minute variations in the precursor ratios profoundly alter the refractive index and film thickness, thereby dictating the macroscopic antireflection performance [30]. Through synergistic control over the micro-morphology and optical properties, we engineered a structurally robust silica matrix exhibiting both sub-nanometer smoothness and an exceptional peak transmittance of 97.4%. This establishes an optimal, high-transmittance “optical host” for the subsequent mesoporous confinement of fluorescent guests.

### 2.3. Structural Integrity of the Mesoporous Matrix and Morphological Stability Post-Functionalization

**Figure 3:**
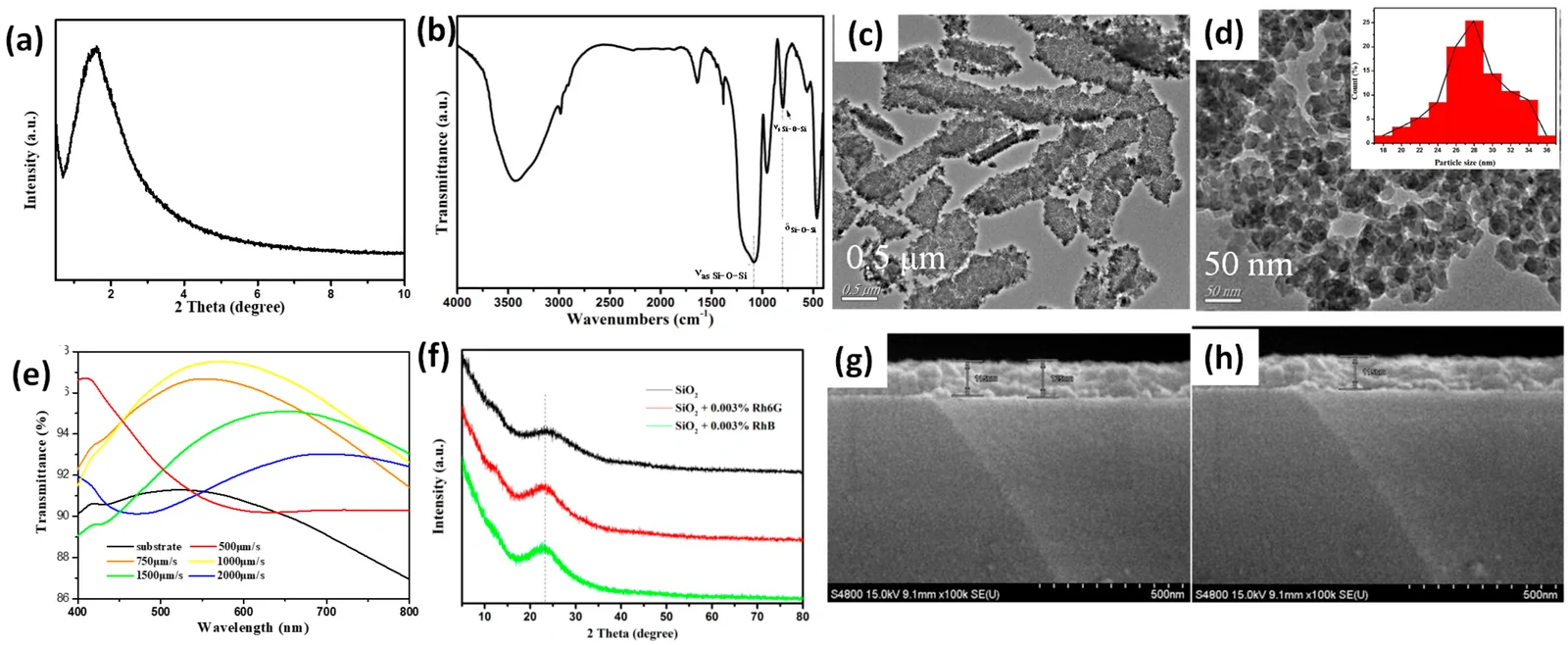
Structural characterization of the mesoporous silica aerogel matrix and its morphological stability after fluorescent functionalization. (**a**) Small-angle X-ray diffraction (SAXRD) pattern and (**b**) FTIR spectrum of the pure silica aerogel film. (**c**) Low-magnification TEM image demonstrating the large-area macroscopic structural uniformity of the matrix. (**d**) High-magnification TEM image revealing the interconnected mesoporous network and sub-nanometer framework. (**e**) UV-Vis transmittance spectra of the pure films at varying draw velocities. (**f**) XRD patterns comparing the pure and dye-doped silica films. Cross-sectional SEM images of the fluorescent films doped with (**g**) Rh6G and (**h**) RhB.

As the “host” for luminescent guests, the pure silica aerogel matrix must possess sufficient structural rigidity to accommodate organic dyes while simultaneously maintaining the ultra-high optical transmittance of the films. Small-angle XRD (SAXRD, Figure 3a) patterns clearly indicate their mesoporous characteristics. Furthermore, the Fourier-transform infrared (FTIR) spectrum (Figure 3b) confirms the successful formation of the robust siloxane framework. The prominent absorption peaks located at 1086 cm^−1^, 806 cm^−1^, and 462 cm^−1^ are assigned to the asymmetric stretching, symmetric stretching, and bending vibrations of the Si–O–Si bonds, respectively. This provides direct molecular-level evidence for the highly cross-linked amorphous silica network, ensuring sufficient structural rigidity to accommodate the organic dyes [31].

The morphological features of the pristine silica aerogel matrix were comprehensively characterized across different length scales to evaluate its suitability as an optical host. Figure 3c presents a low-magnification macroscopic overview of the aerogel film. As observed, the optimized matrix exhibits excellent large-area structural uniformity and continuity, completely devoid of macroscopic phase separation, cracks, or large-scale aggregate formations. This macro-uniformity is a prerequisite for ensuring the ultra-transparency of the optical films.

To further elucidate the internal nanoscale architecture essential for the subsequent guest confinement, high-magnification imaging was conducted (Figure 3d). The high-resolution observation explicitly reveals a typical highly interconnected mesoporous network assembled from uniform amorphous silica nanoparticles (with sizes concentrated at 25–31 nm, as shown in the inset). This robust, sub-nanometer three-dimensional skeleton intrinsically provides abundant and rigid “optical containers” (mesoporous channels), which are critical for the deep encapsulation and spatial isolation of the organic fluorescent dyes. Macroscopically, when the dip-coating withdrawal speed is optimized to 1000 μm/s, the pure film achieves an exceptional optical transmittance of 97.4% (Figure 3e). More importantly, upon the introduction of Rh6G and RhB dyes, the XRD patterns (Figure 3f) exhibit no observable shifts. Concurrently, the cross-sectional SEM images (Figure 3g,h) demonstrate that the dye-doped network remains dense and uniform, preserving a consistent thickness of 110–120 nm.

Mechanistically, the exceptional peak transmittance of 97.4% can be quantitatively supported by the quarter-wavelength antireflection optical model (*n* × *d* = *λ*/4). Based on the cross-sectional SEM observations, the physical thickness (*d*) of the optimized silica network is approximately 110–120 nm. Considering the typical effective refractive index (*n*) of highly porous silica aerogels (~1.15–1.20), the theoretical optimal antireflection wavelength (*λ*) is calculated to be in the range of 504–576 nm. This quantitative optical consistency confirms that the synthesized matrix material acts as a structurally ideal ‘optical host’ for the subsequent guest encapsulation [32]. Furthermore, the comparative structural data before and after functionalization (XRD and SEM) provide compelling evidence that the dye molecules are incorporated into the mesopores via “guest filling” without disrupting the amorphous cross-linked state of the intrinsic Si–O–Si skeleton. This non-destructive skeletal compatibility establishes a robust foundation, prompting us to delve further into the nanoscale to investigate the specific mesoporous encapsulation behaviors of the different dyes.

To clearly visualize the nanoscale surface and internal architectures post-functionalization, newly acquired high-resolution SEM characterization was performed (Figure S3). The top-view SEM image of the RhB-doped film (Figure S3a) explicitly displays a highly uniform and densely packed spherical nanoparticle network, indicative of excellent host–guest compatibility. Its corresponding cross-sectional view (Figure S3b) confirms a continuous, defect-free internal structure. In stark contrast, the surface morphology of the Rh6G-doped film (Figure S3c) exhibits a relatively rougher and more distinctly porous texture, despite maintaining a continuous cross-sectional structure (Figure S3d). This critical visual contrast in surface densification perfectly corroborates the aforementioned AFM and BET results. It provides direct macroscopic evidence that the highly polar RhB molecules achieve superior interfacial integration and tighter mesoporous confinement within the silica matrix compared to Rh6G.

### 2.4. Mesoporous Confinement Behavior and Interfacial Compatibility of the Dyes

To elucidate the confinement discrepancies between the structurally distinct xanthene dyes (Rh6G and RhB) within the aerogel network, we conducted comprehensive parallel interfacial characterization of the functionalized films.

**Figure 4:**
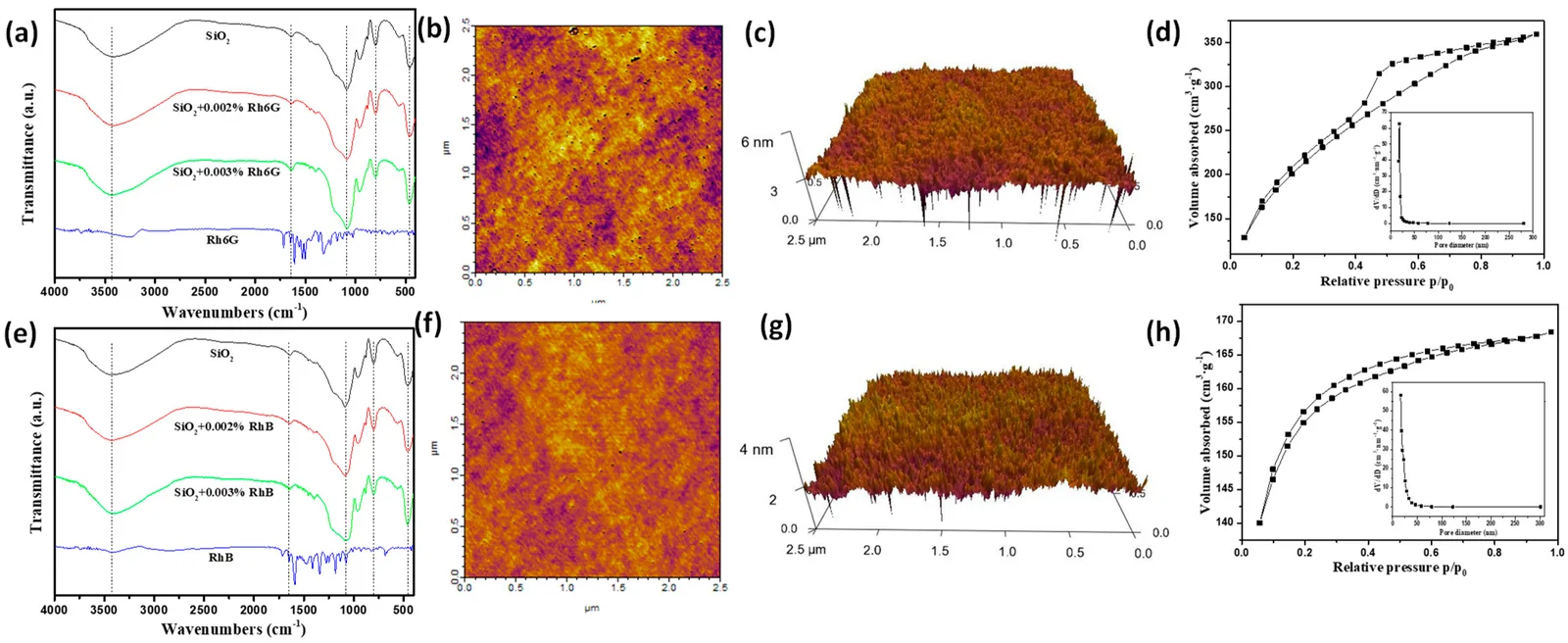
Structural and morphological evolution of the fluorescent functionalized films. FTIR spectra of the silica aerogel films doped with varying contents of (**a**) Rh6G and (**e**) RhB. Corresponding 2D and 3D AFM topographic images of the (**b**,**c**) Rh6G-doped and (**f**,**g**) RhB-doped films. Nitrogen adsorption–desorption isotherms and pore size distributions (insets) for the (**d**) Rh6G-doped and (**h**) RhB-doped films.

FTIR spectra (Figure 4a,e) confirm that the characteristic absorption peaks of the siloxane skeleton (e.g., at 1086 cm^−1^) are robustly preserved irrespective of the dopant dye [33]. However, pronounced differentiations emerge in the morphology and pore structure. The absence of sharp new diffraction peaks and the preservation of the Si–O–Si skeleton bands after dye loading provide compelling evidence that the Rh6G and RhB molecules are successfully encapsulated within the mesopores via physical confinement, without disrupting the intrinsic amorphous network of the silica matrix. AFM (Figure 4b,c vs. Figure 4f,g) reveals that the RhB-doped film exhibits significantly lower surface roughness (RMS = 280.5 pm) compared to the Rh6G system (RMS = 392.3 pm).

**Figure 5:**
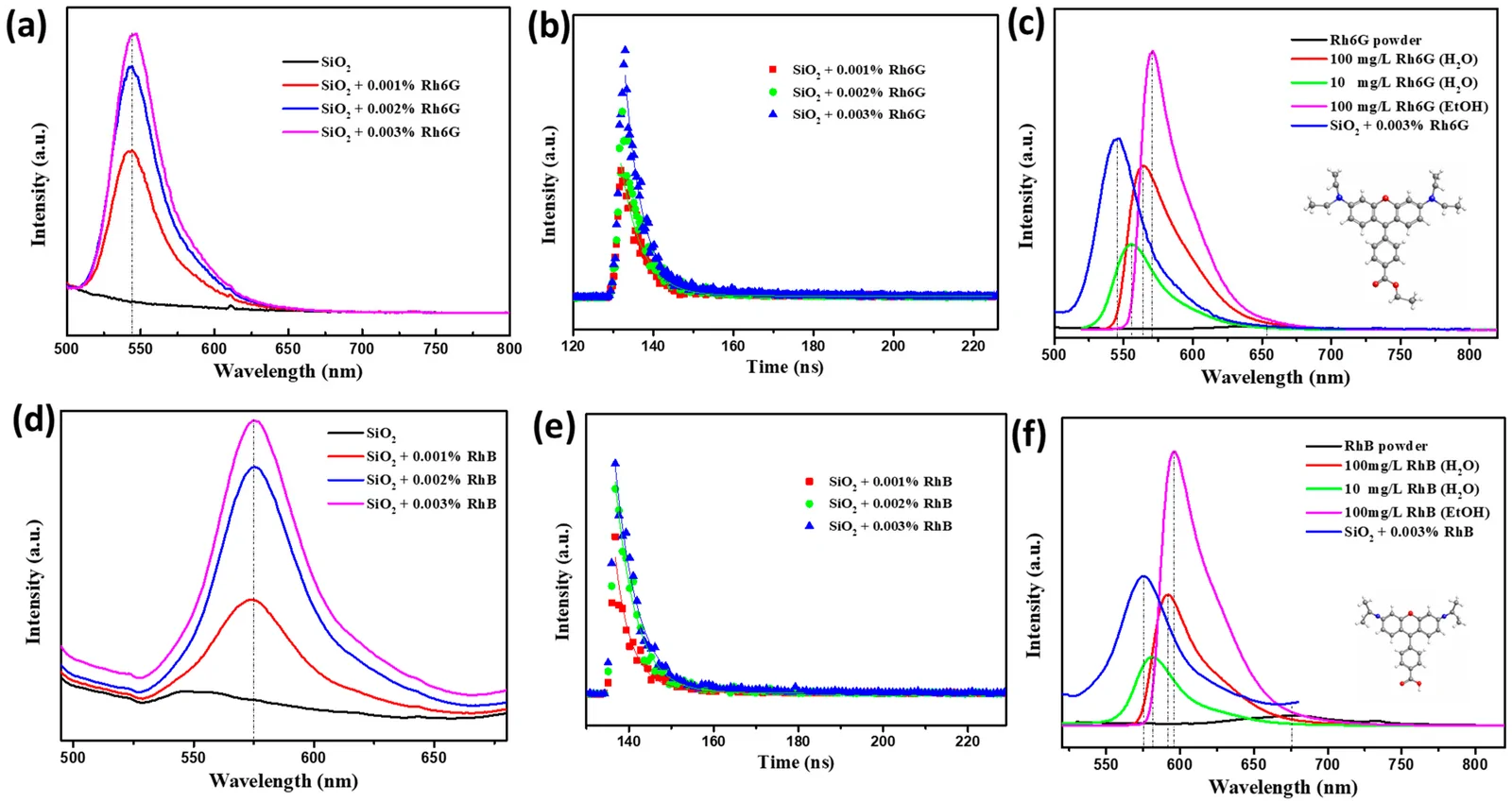
Photoluminescence properties and matrix-induced emission blue shift in the fluorescent aerogel films. Steady-state PL spectra of silica aerogel films doped with varying contents of (**a**) Rh6G and (**d**) RhB. Time-resolved transient fluorescence decay curves of (**b**) Rh6G- and (**e**) RhB-doped films. PL spectra comparison of (**c**) Rh6G and (**f**) RhB across different states (solid powder, solutions, and encapsulated gel films).

To quantitatively evaluate the actual impact of dye incorporation on the internal architecture, nitrogen adsorption–desorption measurements were first performed on the undoped pure silica aerogel film (Figure S1). The pristine matrix exhibited a typical Type IV isotherm with a massive specific surface area of 869.7 m^2^/g and a high pore volume of 0.136 cm^3^/g, establishing an ideal “optical host” with abundant mesoporous voids. The disparities in the nitrogen adsorption–desorption isotherms (Figure 4d vs. Figure 4h) are even more drastic: the RhB system displays an H4-type hysteresis loop [34], accompanied by a precipitous decline in both specific surface area and pore volume (515.8 m^2^/g and 0.019 cm^3^/g, respectively) relative to the Rh6G system (756.8 m^2^/g and 0.027 cm^3^/g, respectively). As explicitly depicted in their chemical structures (Figure 1 and Figure 5), RhB possesses a free, highly polar carboxyl group (–COOH), whereas the corresponding position in Rh6G is occupied by a sterically hindered ester group. This fundamental structural discrepancy dictates their confinement behaviors.

The sharp reduction in pore volume and the evolution of the hysteresis loop from an “ink-bottle” shape (H2) to a “slit-like” geometry (H4) provide compelling evidence for the deep penetration of the dyes into the mesopores [35]. Compared to Rh6G, the RhB molecules are evidently embedded more deeply and tightly within the silica nanochannels. This can be attributed to the stronger polarity or more conformal spatial configuration of the RhB molecules within the matrix pore walls, thereby achieving a highly efficient host–guest confinement [36,37]. This exceptional physical confinement not only refines the macroscopic surface smoothness but also constructs a robust protective barrier for the dye molecules at the microscale, which directly dictates their ultimate photophysical properties.

To quantitatively verify the actual dye incorporation and ensure the complete removal of any non-confined, physically adsorbed surface molecules, the as-prepared functionalized films were subjected to rigorous solvent washing. Subsequently, UV-Vis absorption spectroscopy was performed on the residual washing solutions to evaluate the leaching fraction (Figure S2). Crucially, the spectra exhibited no detectable characteristic absorption peaks corresponding to either RhB (Figure S2a) or Rh6G (Figure S2b). This negligible dye leaching confirms an exceptionally high host–guest encapsulation efficiency (approaching 100%). Consequently, the actual dye loading tightly matches the nominally introduced ultra-low concentration of 0.001–0.003 wt%. This near-perfect retention provides robust quantitative evidence that the xanthene dyes are not merely deposited on the macroscopic exterior, but are firmly and deeply confined within the mesoporous nanochannels, thereby setting a reliable compositional foundation for the subsequent photophysical dynamics.

### 2.5. Photoluminescence Dynamics and Matrix-Induced Emission Blue Shift Mechanism

The successful physical confinement of dye molecules must ultimately translate into enhanced macroscopic optical performance. Organic fluorophores typically suffer from severe aggregation-caused quenching (ACQ) in the solid state; however, the isolation effect provided by the rigid mesoporous channels effectively circumvents this limitation [38,39]. Steady-state photoluminescence (PL) spectra (Figure 5a,d) demonstrate that within the optimal doping concentration range (0.001–0.003%), the functionalized films exhibit robust fluorescence emissions (peaking at ~544 nm for Rh6G and ~575 nm for RhB). The continuous enhancement in PL intensity without the emergence of discernible broad excimer bands indicates the absolute predominance of monomeric dye species within the matrix [40].

Time-resolved transient fluorescence decay measurements (Figure 5b,e) further substantiate this spatial confinement effect. The fluorescence lifetimes of the encapsulated Rh6G and RhB are progressively and significantly prolonged, reaching up to 5.22 ns and 5.36 ns at 0.003% doping concentration, respectively. This substantial lifetime extension confirms that the rigid mesoporous silica network acts as a robust “isolation cabin” [41], successfully suppressing non-radiative transition pathways and intermolecular self-quenching. Specifically, as the doping concentration increases from 0.001 wt% to 0.003 wt%, the lifetimes extend from 4.41 ns to 5.22 ns for Rh6G, and from 2.80 ns to 5.36 ns for RhB. Furthermore, a slightly higher concentration (up to 0.003 wt%) maximizes the host–guest interfacial coupling and the energy transfer efficiency from the silica matrix to the dyes before reaching the ACQ threshold.

More intriguingly, a comparative analysis of the PL spectra across solid powder, solution, and aerogel film states (Figure 5c,f) reveals a pronounced blue shift in the emission peaks for both dyes when incorporated into the aerogel matrix. For instance, the emission peak of Rh6G shifts drastically from 651 nm (solid powder) and 564 nm (aqueous solution) to 547 nm in the confined silica matrix. 

Meanwhile, we attribute this distinct matrix-driven blue shift to the intense host–guest interfacial interactions. The mesoporous silica pore walls are densely decorated with polar silanol groups (–Si–OH) and highly electronegative oxygen ions (–Si–O^−^) [42]. When the Rh6G/RhB molecules, characterized by their xanthene-conjugated aromatic rings, are forcibly confined within these polar nanopores, robust hydrogen-bonding networks and electrostatic interactions emerge at the organic-inorganic interface [43]. This localized interfacial electric field profoundly alters the *π*-electron cloud distribution around the highly electronegative oxygen atoms of the xanthene rings, subsequently enlarging the HOMO-LUMO gap. Consequently, the excited-state energy (Δ*E*) of the dye molecules is elevated [44]. Upon electronic transition back to the ground state, higher-energy photons are emitted, macroscopically manifesting as the observed blue shift [45]. This compelling mechanistic insight not only validates the successful construction of the highly transparent, functionalized matrix but also provides a profound understanding of inorganic-organic interfacial coupling within confined gel networks.

### 2.6. Interfacial Interactions and Mesoporous Confinement Mechanism

**Figure 6:**
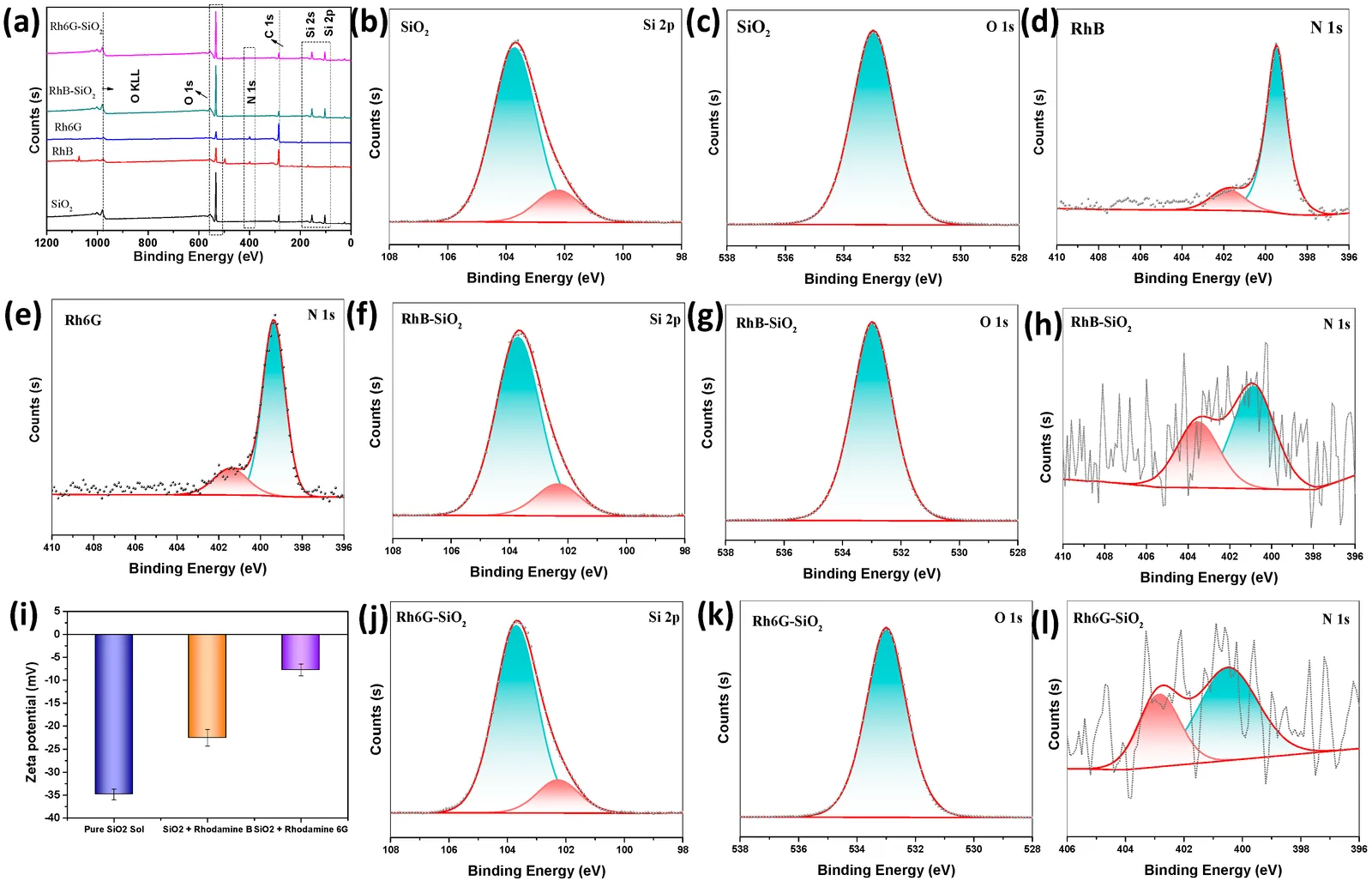
Quantitative characterization of the interfacial host–guest interactions and mesoporous confinement mechanisms. (**a**) XPS survey spectra of the pure silica aerogel, free dyes (Rh6G, RhB), and the heavily dye-doped silica aerogel analogs. High-resolution (**b**,**f**,**j**) Si 2p and (**c**,**g**,**k**) O 1s XPS spectra of pure SiO_2_, RhB-SiO_2_, and Rh6G-SiO_2_, respectively. High-resolution N 1s spectra of (**d**) pure RhB, (**h**) confined RhB in SiO_2_, (**e**) pure Rh6G, and (**l**) confined Rh6G in SiO_2_. (**i**) Zeta potentials of the pristine silica sol and the dye-doped composite sols. The fitted peaks shaded in green and red indicate the binding energy positions of the different valence states.

To explicitly elucidate the micro-environmental interfacial interactions and quantitatively validate the mesoporous confinement mechanism, Zeta potential and high-resolution X-ray photoelectron spectroscopy (XPS) analyses were conducted on the heavily dye-doped silica analogs (Figure 6). 

Macroscopically, the pristine silica sol exhibited a highly negative Zeta potential of −35.2 mV (Figure 6i), intrinsically originating from the abundant highly electronegative oxygen ions (–Si–O^−^) and deprotonated silanol groups on the mesoporous walls. Upon the incorporation of the fluorescent guests, the Zeta potentials shifted significantly to −8.1 mV for the Rh6G-SiO_2_ system and −22.5 mV for the RhB-SiO_2_ system. This pronounced macroscopic charge neutralization provides unequivocal experimental evidence that both positively charged xanthene dyes are successfully confined within the silica network, driven by intense interfacial electrostatic attractions [46,47].

Microscopically, high-resolution XPS provided deeper insights into the host–guest coupling. Compared to their free states, the O 1s and N 1s core-level spectra of the confined dyes exhibited distinct positive shifts towards higher binding energies (Figure 6c–e,g,h,k,l). Notably, the N 1s peak of the RhB-SiO_2_ system demonstrated a significantly larger positive shift than that of the Rh6G-SiO_2_ system. This divergent electronic perturbation perfectly corroborates the Zeta potential findings and elucidates their distinct confinement pathways. Rh6G, lacking strong hydrogen-bonding capabilities due to its sterically hindered ester group, relies almost exclusively on pure electrostatic neutralization (resulting in the massive +27.1 mV Zeta shift). In contrast, RhB possesses a free, highly polar carboxyl group (–COOH), which acts as a robust secondary anchor to form extensive hydrogen-bonding networks with the surface silanol groups. This specific binding firmly pulls the RhB molecule into closer proximity to the electronegative silica matrix, subjecting its conjugated *π*-electron system to a much more intense polarizing interfacial electric field. Consequently, this leads to a stronger depletion of the electron cloud density around the nitrogen atoms in RhB, generating a more pronounced N 1s binding energy shift [48].

Collectively, these spectroscopic and electrokinetic results robustly establish that RhB utilizes a synergistic “electrostatic and hydrogen-bonding” dual-confinement mechanism. This dual mechanism flawlessly explains the superior morphological smoothness, the drastically reduced BET pore volume, and the pronounced matrix-induced emission blue shift observed in the RhB-doped aerogel films.

### 2.7. Proposed Mechanism of the Matrix-Induced Emission Blue Shift

**Figure 7:**
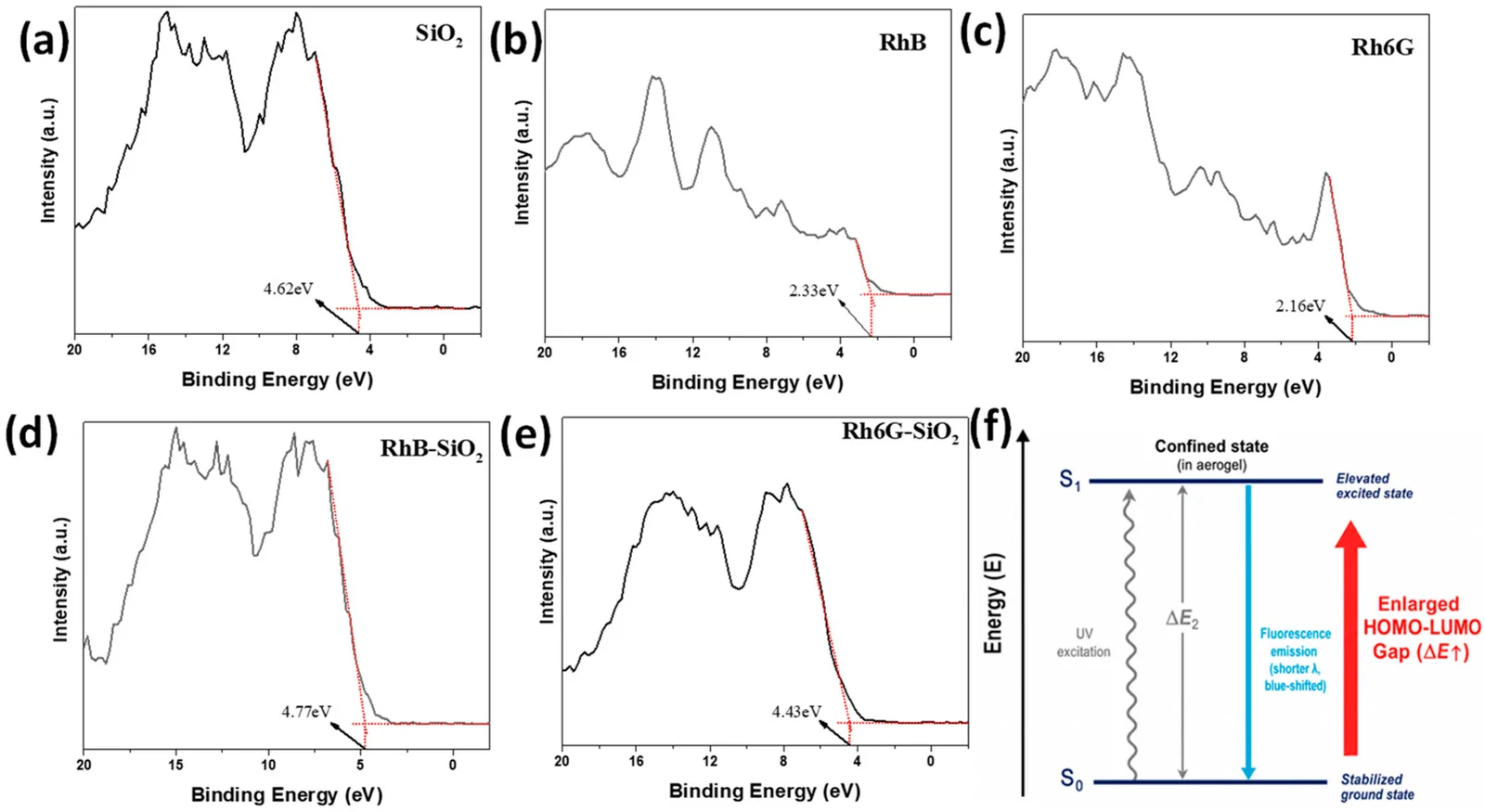
Direct spectroscopic evidence of the enlarged energy gap and the proposed matrix-induced emission blue shift mechanism. Valence band X-ray photoelectron spectroscopy (VB-XPS) spectra of (**a**) pure SiO_2_ aerogel matrix, (**b**) free RhB powder, (**c**) free Rh6G powder, (**d**) confined RhB-SiO_2_ complex, and (**e**) confined Rh6G-SiO_2_ complex. (**f**) Jablonski energy level diagram elucidating the π-electron cloud redistribution, which effectively stabilizes the ground state (S_0_), elevates the lowest excited singlet state (S_1_), and significantly enlarges the HOMO-LUMO energy gap (ΔE).

To fundamentally elucidate the micro-environmental origins of the macroscopic emission blue shift and investigate the altered photophysical dynamics, valence band X-ray photoelectron spectroscopy (VB-XPS) was conducted on the heavily doped analogs. VB-XPS provides robust ground-state spectroscopic evidence for formulating a reliable working hypothesis (Figure 7).

As presented in Figure 7a–e, the valence band maximum (VBM), which precisely corresponds to the highest occupied molecular orbital (HOMO) level of the chromophores, was directly measured. It is crucial to evaluate the electronic perturbation by comparing the confined dyes with their respective free powder states. For the free Rh6G and RhB powders, the VBMs were located at relatively shallow binding energies of 2.16 eV and 2.33 eV, respectively.

However, upon mesoporous encapsulation, the VBM of the confined Rh6G-SiO_2_ and RhB-SiO_2_ complexes exhibited a pronounced shift toward much deeper binding energies of 4.43 eV and 4.77 eV, respectively. This massive positive shift (+2.27 eV for Rh6G and +2.44 eV for RhB) quantitatively demonstrates that the intense interfacial electrostatic and hydrogen-bonding interactions strongly stabilize the ground state (*S*_0_) of the dye molecules. Notably, the more significant downward shift in the HOMO level in the RhB system (+2.44 eV) perfectly aligns with our previous XPS and Zeta potential analyses, further verifying that the highly polar –COOH group of RhB induces a stronger specific “electrostatic and hydrogen-bonding” dual-confinement, leading to a more stabilized ground state.

Based on these solid ground-state experimental observations, we propose a modified Jablonski energy level diagram to hypothesize the matrix-induced blue shift mechanism (Figure 7f). Driven by the robust interfacial coupling, the ground state (S_0_) is highly stabilized. Concurrently, the highly electronegative oxygen ions on the pore walls deeply perturb and polarize the conjugated π-electron cloud of the xanthene rings, which elevates the energy of the lowest excited singlet state (S_1_). This synergistic electronic perturbation inevitably results in a significantly enlarged HOMO-LUMO energy gap (ΔE_2_ > ΔE_1_). Consequently, during the relaxation from the elevated excited state back to the stabilized ground state, higher-energy photons are emitted [47]. By explicitly distinguishing our experimentally demonstrated ground-state static evidence from the proposed excited-state photophysical dynamics, this model provides a data-driven, fundamental explanation for the pronounced macroscopic blue shift observed in the photoluminescence spectra.

Upon continuous UV excitation, the electrons transition to this elevated excited state. When they subsequently relax back to the ground state via radiative pathways, photons with higher energy are emitted. Macroscopically, this translates directly into the observed blue shift in the steady-state PL spectra of the aerogel films compared to their free states in powder or solution (Figure 5c,f). Ultimately, this matrix-induced emission shift not only serves as compelling evidence for the successful host–guest integration but also provides a definitive paradigm for utilizing sol–gel-derived mesoporous networks to rationally manipulate the photophysical dynamics of organic fluorophores.

## 3. Conclusions

In summary, we have demonstrated a comprehensive sol–gel strategy to construct ultra-transparent, fluorescent silica aerogel films driven by precise kinetic control and mesoporous confinement. By systematically tailoring the acid/base dual-catalysis kinetics, an optimal amorphous silica network was engineered, achieving sub-nanometer surface smoothness and an exceptional peak visible transmittance. Utilizing this robust “optical container”, xanthene dyes (Rh6G and RhB) were successfully encapsulated within the nanoscale pores. Notably, RhB exhibited superior interfacial integration with the silica matrix, as evidenced by a distinctive H4 hysteresis loop and a significantly reduced pore volume. Crucially, this mesoporous confinement effectively suppressed aggregation-caused quenching, thereby prolonging the fluorescence lifetimes of the encapsulated dyes. Furthermore, we elucidated that the intense electrostatic and hydrogen-bonding interactions between the polar silanol groups/oxygen ions on the pore walls and the dyes’ conjugated rings elevated the excited-state energy, directly inducing a macroscopic matrix-driven emission blue shift. Ultimately, this work not only provides a scalable methodology for fabricating high-performance optical coatings but also establishes a fundamental paradigm for understanding and manipulating host–guest photophysical dynamics within confined gel networks.

## 4. Materials and Methods

### 4.1. Materials and Reagents

Tetraethyl orthosilicate (TEOS, analytical reagent (AR)), anhydrous ethanol (EtOH, AR), oxalic acid dihydrate (H_2_C_2_O_4_·2H_2_O, AR), ammonium hydroxide (NH_3_·H_2_O, AR), trichloromethane (AR), acetone (AR), Rhodamine 6G (Rh6G, AR), and Rhodamine B (RhB, AR) were utilized without further purification. Deionized water was prepared in the laboratory. Commercial glass slides (Sail brand, Yancheng, China, 75.6 mm × 25.4 mm × 1 mm) were utilized as the coating substrates. All chemicals were purchased from National Medicines Co., Ltd. (Shanghai, China) at analytical reagent (AR) grade.

### 4.2. Substrate Cleaning 

To ensure an immaculate surface for uniform film deposition, the glass substrates were subjected to a rigorous cleaning protocol. The slides were sequentially ultrasonicated in trichloromethane, acetone, anhydrous ethanol, and deionized water to thoroughly eliminate organic and inorganic surface contaminants. Following the sequential washing, the substrates were completely dried under a continuous nitrogen stream and stored in a desiccator prior to use.

### 4.3. Preparation of Silica Sol and Fluorescent Functionalization 

Prior to the sol–gel process, specific stock solutions were prepared: a 0.008 M oxalic acid aqueous solution, a 0.5 M ammonia aqueous solution, and 100 mg·L^−1^ dye stock solutions (prepared by dissolving 50 mg of Rh6G or RhB powder in 500 mL of ethanol).

The highly transparent and fluorescent aerogel films were synthesized via an acid/base two-step sol–gel process. Initially, TEOS and EtOH were mixed uniformly, followed by the addition of the 0.008 M oxalic acid solution to trigger the acid-catalyzed hydrolysis. The mixture was stirred for 12 h at room temperature to ensure the comprehensive formation of linear siloxane oligomers. Subsequently, a specific volume of the 0.5 M ammonia solution was introduced and stirred for 10 min to initiate the base-catalyzed condensation, establishing an optimal precursor molar ratio of 1:7:4:0.001:0.003 (TEOS:H_2_O:EtOH:H_2_C_2_O_4_:NH_3_·H_2_O).

For fluorescent functionalization, 0.4–1.2 mL of the Rh6G or RhB ethanolic stock solution was integrated into the sol immediately after ammonia addition, achieving a dye doping concentration of 0.001–0.003 wt%.

### 4.4. Film Deposition

Once the Tyndall effect was visibly observed upon laser irradiation, the fully mixed functional sol was deposited onto the meticulously cleaned glass substrates utilizing a dip-coating machine (SYDC-100, Shanghai Sanyan Technology Co., Ltd., Shanghai, China). The withdrawal speed was strictly controlled at 1000 μm/s. The resulting wet gel films were then subjected to ambient pressure drying to yield the final mesoporous fluorescent silica aerogel films.

### 4.5. Material Characterization

The frame structure and chemical bonding of the aerogel films were determined using a Fourier-transform infrared spectrometer (FTIR, Nicolet NEXUS 470, Thermo Scientific, Waltham, MA, USA, 4000–400 cm^−1^). The intrinsic amorphous and mesoporous characteristics were analyzed via X-ray diffraction and small-angle X-ray scattering (XRD/SAXRD, Bruker D8 ADVANCE, Karlsruhe, Germany). The cross-sectional morphology and thickness of the films were observed using a scanning electron microscope (SEM, S4800, Hitachi, Tokyo, Japan), and the sub-nanometer surface topography was evaluated by atomic force microscopy (AFM, Bruker, Karlsruhe, Germany). Nitrogen adsorption–desorption isotherms were collected at 77 K using a specific surface area and pore size analyzer (Quantachrome NOVA 2000e, Boynton Beach, FL, USA) to calculate the BET surface area and BJH pore size distribution. The macroscopic optical antireflection properties and transmittance were recorded on a UV-Vis spectrophotometer (TU-1810, Beijing Purkinje General Instrument Co., Ltd., 400–800 nm, Beijing, China). Steady-state photoluminescence (PL) spectra and time-resolved transient fluorescence decay lifetimes were measured using a fluorescence spectrophotometer equipped with a time-correlated single-photon counting (TCSPC) module (QuantaMaster 40, Photon Technology International, Birmingham, NJ, USA). The micro-environmental chemical states and elemental compositions of the functionalized matrices were analyzed using an X-ray photoelectron spectrometer (XPS, ESCALAB 250Xi, Thermo Fisher Scientific, Waltham, MA, USA). The macroscopic surface charges and Zeta potentials of the precursor sols were evaluated using a nanoparticle analyzer (Zetasizer Nano ZS90, Malvern Instruments, Malvern, UK).

All quantitative experiments and characterization, including peak transmittance evaluation, were independently repeated on at least three different sample batches (n = 3). The corresponding numerical data are expressed as the mean ± standard deviation (SD) throughout the manuscript. Error bars in the relevant figures represent the standard deviation of these replicate measurements.

## Figures and Tables

**Table 1:** Textural physical properties of silica-based aerogel films.

Parameter	Pure SiO_2_ Film	Rh6G-Doped SiO_2_ Film	RhB-Doped SiO_2_ Film
Surface Roughness RMS (pm)	276.7	392.3	280.5
Relative mean deviation Ra (pm)	198.4	260.4	221.8
Height Range R_max_ (pm)	4.97	6.89	5.79
Film Thickness (nm)	110–120	110–120	110–120
BET Surface Area (m^2^/g)	869.7	756.8	515.8
Pore Volume (cm^3^/g)	0.136	0.027	0.019
Peak Transmittance (%)	97.4 ± 0.3	92.3 ± 0.2	95.1 ± 0.1
Fluorescence Lifetime (ns)	N/A	5.22	5.36

## Data Availability

The original contributions presented in the study are included in the article/Supplementary Material; further inquiries can be directed to the corresponding author.

## Supplementary Materials

The following supporting information can be downloaded at: https://www.mdpi.com/article/10.3390/gels12080676/s1, Figure S1. Nitrogen adsorption–desorption isotherm (a) and pore size distribution (b) for the pure SiO_2_ film. Figure S2. UV spectra of the solution obtained after washing the film samples: (a) Rhodamine B, (b) Rhodamine 6G. Figure S3. High-resolution scanning electron microscopy (SEM) characterizations of the dye-doped silica aerogel films. (a) Top-view surface morphology and (b) cross-sectional view of the RhB-doped SiO_2_ film, demonstrating a highly dense and uniform integration. (c) Top-view surface morphology and (d) cross-sectional view of the Rh6G-doped SiO_2_ film, exhibiting a relatively more porous surface texture. All scale bars are distinctly indicated as 400 nm.
