# Supplementary material for: Mesoporous Confinement of Fluorescent Dyes in Ultra-Transparent Silica Aerogel Films via Tailored Sol–Gel Kinetics
Source: Gels. 2026 Jul 30;12(8):676. doi: 10.3390/gels12080676 (PMC13512029; doi:10.3390/gels12080676)
Supplement: Supplementary file 1 [file gels-12-00676-s001.zip › gels-4473697-supplementary.pdf]

Supplementary data

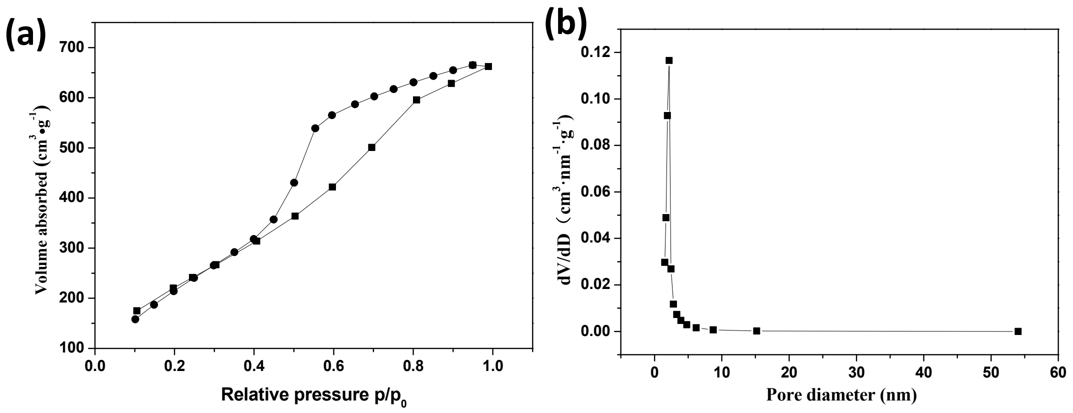

Supplementary Figure S1. Nitrogen adsorption-desorption isotherm (a) and pore size distribution (b) for the pure SiO_2 film.

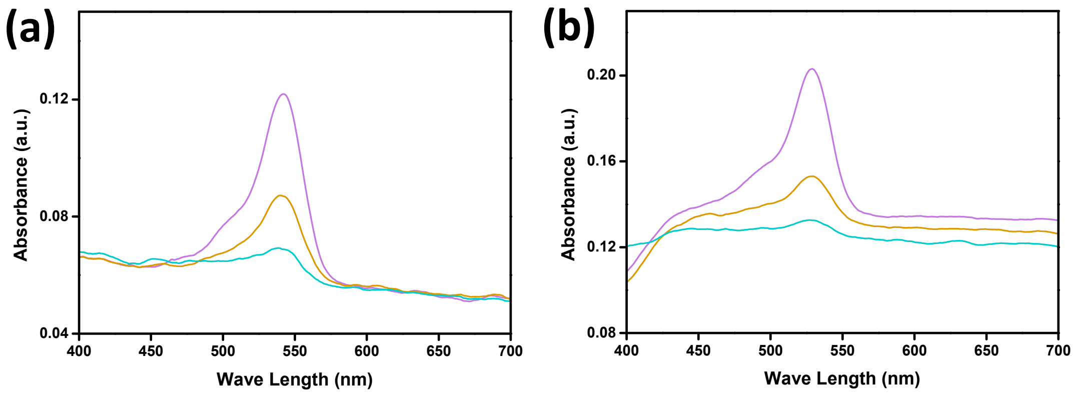

Supplementary Figure S2. UV spectra of the solution obtained after washing the film samples: (a) Rhodamine B, (b) Rhodamine 6G.

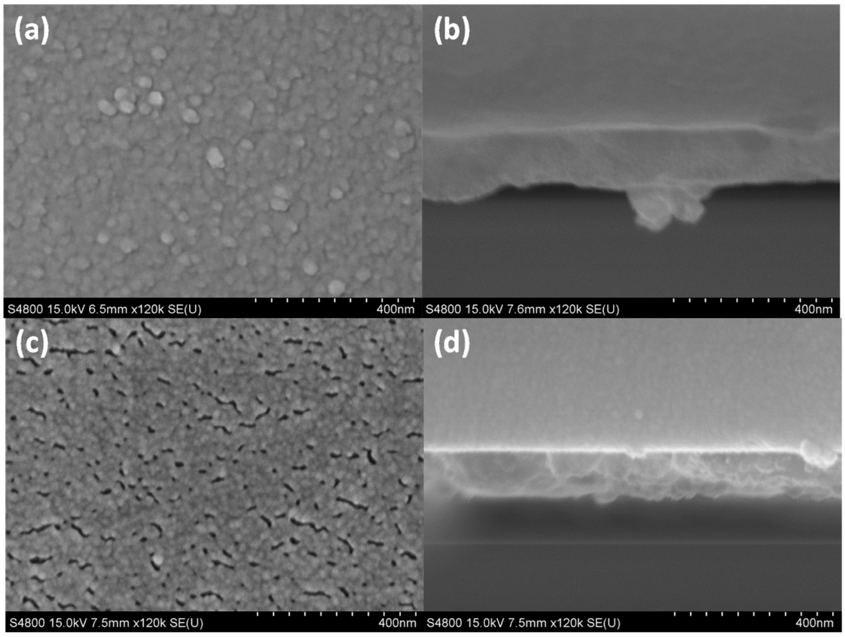

Supplementary Figure S3. High-resolution scanning electron microscopy (SEM) characterizations of the dye-doped silica aerogel films. (a) Top-view surface morphology and (b) cross-sectional view of the RhB-doped SiO_2 film, demonstrating a highly dense and uniform integration. (c) Top-view surface morphology and (d) cross-sectional view of the Rh6G-doped SiO_2 film, exhibiting a relatively more porous surface texture. All scale bars are distinctly indicated as 400 nm.
